# Hierarchical distance-based fuzzy approach to evaluate urban water supply systems in a semi-arid region

**DOI:** 10.1186/s40201-015-0206-y

**Published:** 2015-07-14

**Authors:** Tahereh Sadeghi Yekta, Mohammad Khazaei, Ramin Nabizadeh, Amir Hossein Mahvi, Simin Nasseri, Ahmad Reza Yari

**Affiliations:** Research Center for Environmental Pollutants, Qom University of Medical Sciences, Qom, Iran; Department of Environmental Health Engineering, School of Public Health, Tehran University of Medical Sciences, Poursina St, Keshavarz Blvd, PO BOX: 6446-14155, Tehran, Iran; Center for Solid Waste Research, Institute for Environmental Research, Tehran University of Medical Sciences, Tehran, Iran; Center for Water Quality Research, Institute for Environmental Research, Tehran University of Medical Sciences, Tehran, Iran

**Keywords:** Fuzzy logic, Drinking water, MCDM, Distribution system

## Abstract

Hierarchical distance-based fuzzy multi-criteria group decision making was served as a tool to evaluate the drinking water supply systems of Qom, a semi-arid city located in central part of Iran. A list of aspects consisting of 6 criteria and 35 sub-criteria were evaluated based on a linguistic term set by five decision-makers. Four water supply alternatives including “Public desalinated distribution system”, “PET Bottled Drinking Water”, “Private desalinated water suppliers” and “Household desalinated water units” were assessed based on criteria and sub-criteria.

Data were aggregated and normalized to apply Performance Ratings of Alternatives. Also, the Performance Ratings of Alternatives were aggregated again to achieve the Aggregate Performance Ratings. The weighted distances from ideal solution and anti-ideal solution were calculated after secondary normalization. The proximity of each alternative to the ideal solution was determined as the final step. The alternatives were ranked based on the magnitude of ideal solutions.

Results showed that “Public desalinated distribution system” was the most appropriate alternative to supply the drinking needs of Qom population. Also, “PET Bottled Drinking Water” was the second acceptable option. A novel classification of alternatives to satisfy the drinking water requirements was proposed which is applicable for the other cities located in semi-arid regions of Iran.

The health issues were considered as independent criterion, distinct from the environmental issues. The constraints of high-tech alternatives were also considered regarding to the level of dependency on overseas.

## Introduction

Evaluating the alternatives to satisfy the drinking water demands of societies is a complicated issue that usually should be relied on human judgments. Furthermore, Different criteria should be considered to evaluate the alternatives available for supplying the drinking water needs, especially in populations faced with fresh water scarcity which are relied on brackish water sources [1].

Various methods based on human decision-making have been used to evaluate the alternatives assigned for water supply systems such as Life cycle assessment [2, 3], MCDM approach [4], Five-parametric matrix [5], Multi-criteria decision aid (MCDA) approach [6], and consumer cooperatives [7].

The major concern related to the water supply systems in developing countries is the large scale projects such as trans-basin water transfer [8], and constructing the sophisticated water supply systems which may not be completed on time because of the financial deficiencies or changing in political considerations [9]. So, applying the available water supply systems as the viable alternatives can be helpful to deliver an obvious viewpoint for administrators as well as for the public sector [10]. Also, few studies, worked on evaluating the available alternatives, have drown the hierarchy of aspects directly from the other studies and did not consider the background factors in their intrinsic society which may influence the arrangement of criteria and sub-criteria [4, 7, 11].

This paper outlines a methodology that evaluates the available alternatives to supply drinking water demands of Qom population, a city located in plains fed with brackish aquifers. The evaluation processes are according to a complete package of criteria and sub-criteria.

A simple-minded and well-known method of decision-making is adopted based on fuzzy logic to evaluate the alternatives. The presented method is known as hierarchical distance-based fuzzy multi-criteria group decision making (DBF –MCDM) approach. Applying DBF–MCDM enables the decision-making committee to improve the identification of discrepancies and similarities of their judgments [12]. Also, the DBF–MCDM process justifies both ideal and anti-ideal solutions simultaneously that help the decision-makers to have more obvious judgments [13]. A new arrangement of criteria and sub-criteria to evaluate the drinking water supply alternatives is also adopted using the MCDM method under fuzzy environment.

## Methodology

Various aspects should be considered when a team or organization decides to make a decision among several available alternatives. The decision making process maybe comes more complicated if the number of alternatives and criteria be increased [14]. This section dedicates a short description about the principles of multi-criteria group decision making (MCDM) that is based on fuzzy set theory to resolve the decision making problems on the subject of drinking water supply alternatives.

### Fuzzy sets theory

#### Definition 1

A fuzzy set can be defined as *Ã* = (*X*, *μ*_*Ã*_(*x*)), Where X is the space on which the fuzzy set is defined, and *μ*_*Ã*_(*x*) → [0, 1], *x* ∈ *X*, the membership function of the set [15].

#### Definition 2

**Fig. 1:**
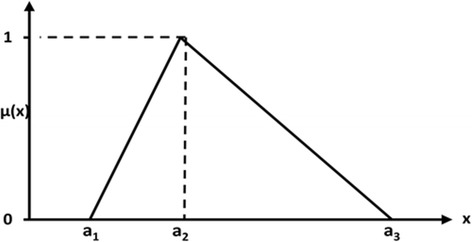
A triangular fuzzy number *Ã*

As shown in Fig 1, a triangular fuzzy number *Ã* can be depicted with a triplet (*a*_1,_*a*_2,_*a*_3_) which its membership function are symbolized as follows [16]:

1 $$ \begin{array}{l}\\ {}{\mu}_{\tilde{A}}(x)\kern0.5em =\left\{\begin{array}{c}\hfill \frac{x-{a}_1}{a_2-{a}_1},{a}_1\kern0.5em \le \kern0.5em x\kern0.5em {a}_2,\hfill \\ {}\hfill \frac{x-{a}_3}{a_2-{a}_3},{a}_2\kern0.5em \le \kern0.5em x\kern0.5em {a}_3,\hfill \\ {}\hfill 0,\kern3.5em  Otherwise.\hfill \end{array}\right.\end{array} $$

Using the triangular fuzzy number is due to its simplicity compare with trapezoid or sigmoid fuzzy numbers and intuitively easy for decision-makers to utilize. Furthermore, modeling according to triangular fuzzy numbers is a competent approach for organizing the decision-making problems [17, 16].

#### Definition 3

A linguistic variable is defined as a kind of variable whose values are expressed in linguistic terms. Because of the imprecise and vague nature of human judgments, it is preferred to express the expert judgments via linguistic terms. The linguistic terms are the study variables with the capability of describing the qualitative data. A linguistic variable comprises an ordinary word or phrase in natural language and so they are representatives of imprecise data whose values are not numbers. In situations that the study has been affected by ill defined or complex variables, a linguistic term can be a useful tool to prepare an approximate characterization [18].

#### Definition 4

The criteria *a*_1_, *a*_2_ …, *a*_3_ are defined as the evaluation tools of each alternative. This assumption must be taken into account that all criteria are relevant for various alternatives. The different alternatives are represented as *A*_1_, *A*_2_ … ., *A*_*m*_ For certain alternative *A*_*i*_, the relative value of criteria *a*_*i*_ is allocated by a rating, identified as r_ij_. Also, the relative importance of a given criterion a_j_ is allocated by a weighting coefficient, denoted as w_j_. So, the alternative A_i_ obtains the weighted average rating as follows:

2 $$ {\overline{r}}_i\kern0.5em =\kern0.5em \frac{{\displaystyle \sum \begin{array}{c}\hfill n\hfill \\ {}\hfill j\kern0.5em =1\hfill \end{array}}{W}_i{r}_{ij}}{{\displaystyle \sum \begin{array}{c}\hfill n\hfill \\ {}\hfill j=1\hfill \end{array}{W}_j}} $$

Comparing and ranking the final ratings ${\overline{r}}_1,{\overline{r}}_2\dots, {\overline{r}}_m$ are performed to judge the relevant values of the different alternatives [14].

#### Definition 5

If *ñ* be considered as a triangular fuzzy number and $$ n\begin{array}{c}\hfill \propto \hfill \\ {}\hfill \ell \hfill \end{array}>\kern0.5em 0,n\begin{array}{c}\hfill \propto \hfill \\ {}\hfill u\hfill \end{array}\le \kern0.5em 1 $$ for ∝ ∈ [0, 1] then *ñ* is called a normalized positive triangular fuzzy number [19].

#### Definition 6

The ideal solution A* = (*r*_1_*, *r*_2_*, …, *r*_n_*) and also the anti-ideal solution *A*^−^ = (*r*_1_^−^, *r*_2_^−^ …, *r*_*n*_^−^) are defined where *r*_j_* = (1, 1, 1) and *r*_*j*_^−^ = (0, 0, 01) for *j* = 1, 2 …, *n* [20].

#### Definition 7

The distance measure ${d}_v\left(\tilde{A},\tilde{B}\right)$ is applied to indicate the distance between the fuzzy numbers *Ã* = (*a*_1_, *a*_2_, *a*_3_) and $\tilde{B}=\left({b}_1,{b}_2,{b}_3\right)$ as follows [21]:

3 $$ {d}_v\left(\tilde{A},\tilde{B}\right)=\frac{1}{2}\left\{ \max \kern0.5em \left(\left|{a}_1-{b}_1\left|,\right|{a}_3-{b}_3\right|\right)+\left|{a}_2-{b}_2\right|\right\} $$

The size of the trapezoidal area is obtained by the distance formula. The larger values of |*a*_1_ − *b*_1_| *or*|*a*_3_ − *b*_3_| are the lower trapezoid base. The values of |*a*_2_ − *a*_2_| determine the upper trapezoid base, and the trapezoid height is equal to one. The Closer triangular numbers $\tilde{A}\kern0.5em  and\kern0.5em \tilde{B}$ the smaller trapezoidal area.

### Hierarchical distance-based fuzzy Multi-criteria group decision making (DBF –MCDM) approach

The fuzzy multi-criteria group decision making approach has the ability of addressing the decision problems including a multi-level hierarchical structure which has been equipped with attributes of qualitative performance [22]. The distance-based fuzzy MCDM approach has been introduced by Karsak (2002) for selecting the technology alternative [23]. The DBF-MCDM is constructed according to the closeness to the ideal alternative concept. Also, DBF-MCDM has the potential of including both crisp and fuzzy data.

Usually, the performance attributes can be organized in multi-level hierarchy when they are in large numbers. The multi-level hierarchy enables the analysis to be done more efficiently.

**Fig. 2:**
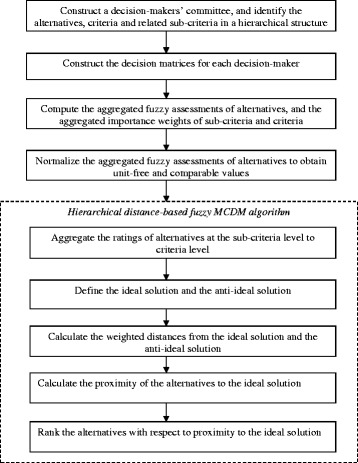
Representation of the distance-based fuzzy MCDM algorithm

Here, a subversion known as “multi-expert” from the algorithm of hierarchical DBF-MCDM which originally introduced by Karsak and Ahiska (2005) and later represented by Dursun (2011.a) is applied. Figure 2 illustrates a brief representation of hierarchical DBF-MCDM approach.

The following successive steps present the hierarchical DBF-MCDM approach implementation:

Step 1. Establish a decision- makers team of *z* experts (*l = 1,2…, z*). Introduce the alternatives, necessary criteria, and attributed sub-criteria.
Step 2. Assemble the decision matrices that comprise the importance weights of criteria and attributed sub-criteria. The decision matrices also, should be included the fuzzy assessments in relation with sub-criteria for each decision-maker.
Step 3. Introduce the mathematical signs used for representation the criteria, sub-criteria, decision makers and alternatives and their relationships as depicted in Table 1.

**Table 1:** Mathematical signs used for representing the equations

Definition	Description
*i* = (1, 2 …, *m*)	Set of alternatives
*j* = (1, 2 …, *n*)	Set of criteria
*k* = (1, 2 …, *p*)	Set of sub-criteria
*l* = (1, 2 …, *z*)	Set of decision makers
${\tilde{X}}_{ijkl}=\left({X}_{ijkl}^1,{X}_{ijkl}^2,{X}_{ijkl}^3\right)$	Alternative *i* attributed to sub-criterion *k* of criterion *j*.
${\tilde{W}}_{jkl}=\left({W}_{jkl}^1,{W}_{jkl}^2,{W}_{jkl}^3\right)$	Importance weight of sub-criterion *k* of criterion *j*.
${\tilde{W}}_{jl}=\left({W}_{jl}^1,{W}_{jl}^2,{W}_{jl}^3\right)$	Importance weight of criterion *j* for the *l*th decision-maker
Step 4. Calculate the aggregated fuzzy assessments of alternatives $\left({\tilde{X}}_{ijkl}\right)$, the aggregated importance weight of sub-criteria $\left({\tilde{W}}_{jkl}\right)$ and the aggregated importance weight of criteria $\left({\tilde{W}}_{jl}\right)$ based on follows:
  4 $$ {\tilde{W}}_j={\displaystyle \sum_{l-1}^z{v}_l}{\tilde{W}}_{jl} $$
  5 $$ {\tilde{W}}_{jk}={\displaystyle \sum_{l-1}^z{v}_l}{\tilde{W}}_{jkl} $$
  6 $$ {\tilde{X}}_{ijk}={\displaystyle \sum_{l-1}^z{v}_l}{\tilde{X}}_{ijkl} $$
  Where *v*_*l*_ ∈ [0, 1] represents weight assigned to the *l*th decision-maker.
  Also, ∑_*l* = 1_^*z*^*v*_*l*_ = 1.
  So, by using above equations, aggregated ratings of alternatives with respect to each sub-criterion $\left({\tilde{X}}_{ijk}\right)$, aggregated importance weights of sub-criteria ${\tilde{W}}_{jk}$ and aggregated importance weights of criteria $\left({\tilde{W}}_j\right)$ can be computed as (*X*_*ijk*,_^1^*X*_*ijk*,_^2^*X*_*ijk*,_^3^), (*W*_*jkl*,_^1^*W*_*jkl*,_^2^*W*_*jk*,_^3^) and (*W*_*j*,_^1^*W*_*j*,_^2^*W*_*j*,_^3^) respectively.
Step 5. To obtain the unit-free and comparable sub-criteria values, the aggregated decision matrix resulted from step 4 should be normalized. Among various methods used for data normalization [24, 17] a linear scale transformation is selected. Based on this approach, first the sub-criteria are categorized in two groups known as benefit-related (BR) and cost related (CR) ones as identified in Fig. 3. Then, the linear scale transformation is used for data normalization as follows:

**Fig. 3:**
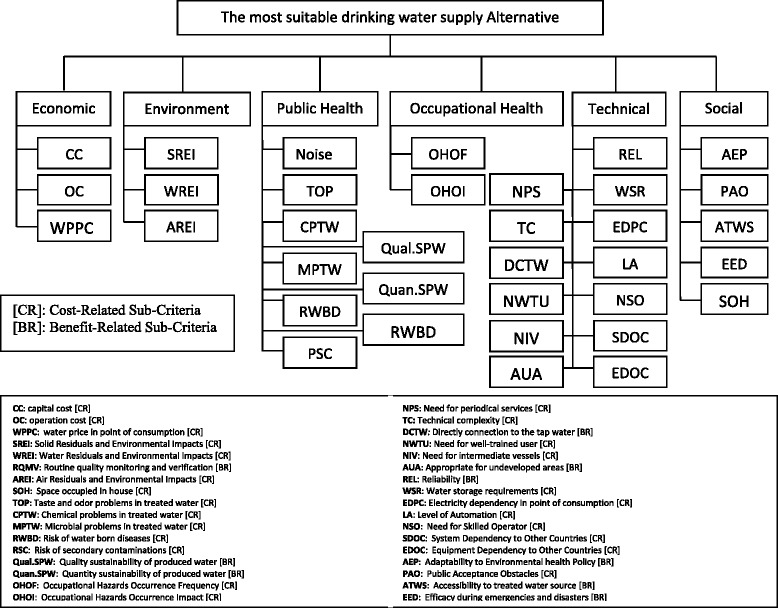
Hierarchical structure of the problem and identifying the CR and CB nature of criteria and sub-criteria

  7 $$ \begin{array}{l}{\tilde{r}}_{ijk}=\left({r}_{ijk}^1,{r}_{ijk}^2,{r}_{ijk}^3\right)\\ {}=\left\{\begin{array}{c}\hfill \left(\frac{x_{ijk}^1-{x}_{jk}^{-}}{x_{jk}^{*}-{x}_{jk}^{-}},\frac{x_{ijk}^2-{x}_{jk}^{-}}{x_{jk}^{*}-{x}_{jk}^{-}},\frac{x_{ijk}^3-{x}_{jk}^{-}}{x_{jk}^{*}-{x}_{jk}^{-}}\right),\kern0.5em k\in \kern0.5em \mathrm{B}{\mathrm{R}}_j;\kern0.5em i=1,2\dots, m;\kern0.5em j=1,2\dots, n\hfill \\ {}\hfill \left(\frac{x_{jk}^{*}-{x}_{ijk}^3}{x_{jk}^{*}-{x}_{jk}^{-}},\frac{x_{jk}^{*}-}{x_{jk}^{*}-},\frac{x_{ijk}^2}{x_{jk}^{-}},\frac{x_{jk}^{*}-{x}_{ijk}^1}{x_{jk}^{*}-{x}_{jk}^{-}}\right),k\in \kern0.5em C{\mathrm{R}}_j;\kern0.5em i=1,2\dots, m;\kern0.5em J=1,2\dots, n\hfill \end{array}\right.\end{array} $$
  Where, ${\tilde{r}}_{ijk}$ is the normalized value of ${\tilde{x}}_{ijk}$, *x*_jk_^*^ denotes max_*i*_*x*_*ijk*_^3^ and *x*_*jk*_^−^ is min_*i*_*x*_*ijk*_^1^BR_j_ is the set of benefit-related sub-criteria of criterion *j* for which the higher the efficiency value the more performance of it and CR_j_ is the sets of cost-related sub-criteria of criterion *j* for which the higher the efficiency value the less preference of it. Also, *m* identifies the number of alternatives and *n* denotes the number of criteria.
Step 6. The performance ratings of alternatives at the sub-criteria stage to criteria stage should be aggregated to compute the aggregate performance ratings (APRs) as follows:
  8 $$ {\tilde{y}}_{ij}=\left({y}_{ij}^1,{y}_{ij}^2,{y}_{ij}^{31}\right)=\frac{{\displaystyle {\sum}_{k=1}^p\kern0.5em {\tilde{w}}_{jk}\otimes}\kern0.5em {\tilde{r}}_{ijk}}{{\displaystyle {\sum}_{k=1}^p{\tilde{w}}_{jk}}},i=1,2\dots, m;j=1,2\dots, n $$
  Where, *ỹ*_*ij*_ is served as the APR of alternative *i* in relation with criterion j. It should be added that ⊗ is the multiplication operator in fuzzy logic.
Step 7. The APRs are normalized at criteria stage with linear normalization method again. Based on this approach and as can be recognized from the following equation, the best results acquire the value equal 1 and the worst ones obtain the value equal 0.
  9 $$ {\overset{\tilde{\mathit{\hbox{'}}}}{y}}_{ij}=\left({\overset{\tilde{\mathit{\hbox{'}}}}{y}}_{ij}^1,{\overset{\tilde{\mathit{\hbox{'}}}}{y}}_{ij}^2,{\overset{\tilde{\mathit{\hbox{'}}}}{y}}_{ij}^3\right)=\left(\frac{y_{ij}^1-{y}_j^{-}}{y_j^{*}-{y}_j^{-}},\frac{y_{ij}^2-{y}_j^{-}}{y_j^{*}-{y}_j^{-}},\frac{y_{ij}^3-{y}_j^{-}}{y_j^{*}-{y}_j^{-}}\right),\kern1em i=1,2\kern0.5em \dots, \kern0.5em \mathrm{m};\kern0.5em j=1,2\kern0.5em \dots, \kern0.5em \mathrm{n} $$
  Where, ${\overset{\tilde{\mathit{\hbox{'}}}}{y}}_{ij}$ is the normalized APR of alternative *i* with respect to criterion *j. y*_j_^*^ = max_*i*_*y*_*ij*_^3^ and *y*_*j*_^−^ = min_*i*_*y*_*ij*_^1^.
Step 8. The weighted distances (WDs) from ideal solution and anti-ideal solution may be represented as *D*_i_^*^ and *D*_*i*_^−^ respectively. The value of WD for each alternative can be computed as follows:
  10 $$ o{D}_i^{*}={\displaystyle \sum_{j=1}^n\frac{1}{2}}\left\{ \max \left({\tilde{w}}_j^1\left|{\overset{\tilde{\mathit{\hbox{'}}}}{y}}_{ij}^1-1\right|,{\tilde{w}}_j^3\Big|{\overset{\tilde{\mathit{\hbox{'}}}}{y}}_{ij}^3-1\right)+{\tilde{w}}_j^2\left|{\overset{\tilde{\mathit{\hbox{'}}}}{y}}_{ij}^2-1\right|\right\},\kern0.5em \mathrm{i}=1,2\kern0.5em \dots, \kern0.5em \mathrm{m} $$
  11 $$ {D}_i^{-}={\displaystyle \sum_{j=1}^n\frac{1}{2}}\left\{ \max \left({\tilde{w}}_j^i\left|{\overset{\tilde{\mathit{\hbox{'}}}}{y}}_{ij}^1-0\right|,{\tilde{w}}_j^3\left|{\overset{\tilde{\mathit{\hbox{'}}}}{y}}_{ij}^3-0\right|\right)+{\tilde{w}}_j^2\left|{\overset{\tilde{\mathit{\hbox{'}}}}{y}}_{ij}^2-0\right|\right\},\kern2em \mathrm{i}=1,2\dots, \mathrm{m} $$
Step 9. The proximity of the alternatives to the ideal solution is represented with *Ω*_i_^*^ and can be calculated as follows:
  12 $$ {\Omega}_i^{*}=\frac{D_i^{-}}{D_i^{*}+{D}_i^{-}},\kern3em \mathrm{i}=1,2\dots, \mathrm{m}. $$
  By using the *Ω*_i_^*^ concept, the distances from ideal and anti-ideal solutions are computed.
If the results of *Ω*_i_^*^ are sorted from largest to the smallest values, the best alternative is one which has obtained the highest *Ω*_i_^*^ value and therefore is located in the top of the descending ranking of alternatives.

### Study area

**Fig. 4:**
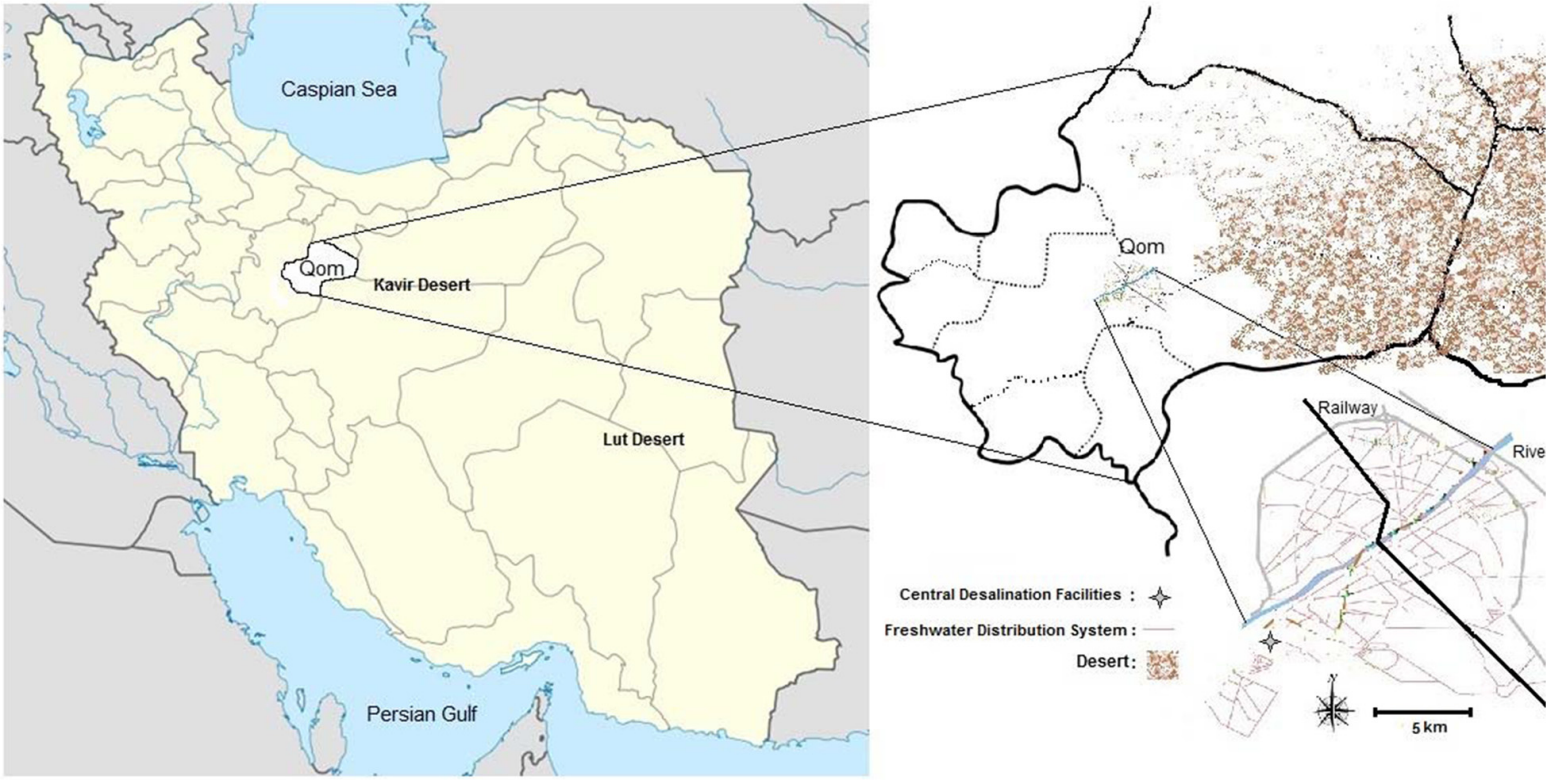
A schematic view of Qom central desalination facilities and its freshwater distribution system

As shown in Fig. 4, Qom province has been located in central part of Iran. Qom is the only city of province and has the population more than 1 million permanent inhabitants. Qom is the second city in Iran after Mashhad as a pilgrimage center [25], so its population has noticeable annually fluctuations because of religious tourists reception [26].

Qom province has low annual precipitation and also salty marls are prevalence geological structures [27] in its plains. Consequently, like the other cities located in central part of Iran, Qom population has engaged with both water quality and quantity crisis [28, 29]. Local water sources of Qom which are flowed in public salty distribution system (PSDS) contain relatively high levels of salt and are considered only for non-drinking purposes. Dissolved solids concentration (TDS) of surface water and groundwater sources of the province is around 1800 and 4500 mg/L, respectively. To improve the quality of these brackish water sources for drinking demands, some programs have been carried out since past decades, such as Public desalinated distribution system (PDDS), Private desalinated water suppliers (PDWS), and Household desalinated water units (HDWU) [29].

### Evaluating drinking water supply alternatives using DBF –MCDM approach

The following methods were considered as capable alternatives to supply the drinking water demands of Qom:

- A_1_: Public desalinated distribution system (PDDS)
- A_2_: PET Bottled Drinking Water (PBDW)
- A_3_: Private desalinated water suppliers (PDWS)
- A_4_: Household desalinated water units (HDWU)

Six and 35 evaluation criteria and sub-criteria were defined, respectively which illustrated in Fig. 3. Also, sub-criteria were classified to Cost-Related and Beneficial-Related groups. The benefit-related sub-criteria are those for which the higher the performance value the more its preference, and the cost-related sub-criteria are considered as sub-criteria for which the higher the performance value the less its preference (Fig 3).

**Fig. 5:**
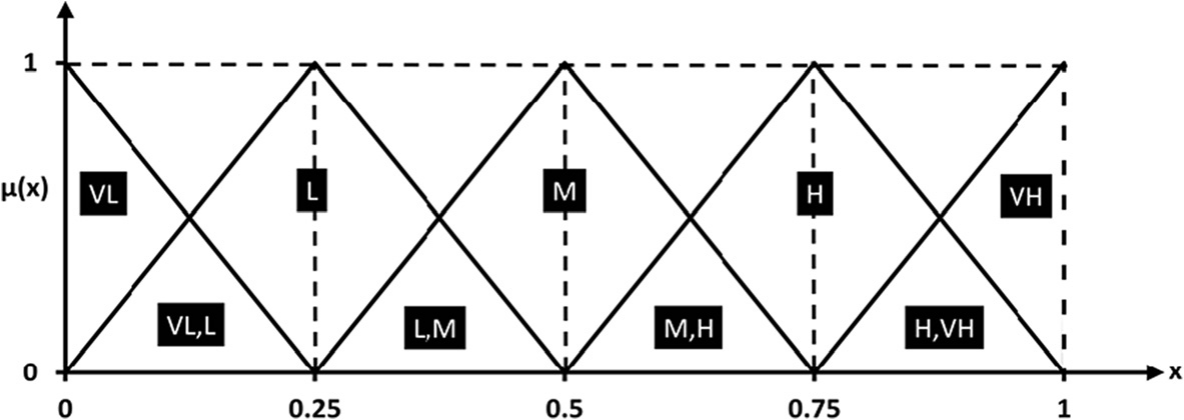
Linguistic term set in fuzzy depiction

The evaluation was performed by a team of five decision-makers which are identified as *DM*_1_, *DM*_2_, *DM*_3_, *DM*_4_ and *DM*_5_. DM_1_ is a professor of environmental health engineering. DM_2_ is a technical advisor specialized in water desalination facilities, DM_3_ is a professor in epidemiology, DM_4_ is a water treatment expert from Qom Water and Sewage Company (QWSC), and DM_5_ is a socio-economic advisor specialized in urban water management. Decision-makers used the linguistic term set shown in Table 2 which also has illustrated as a fuzzy triangular depiction in Fig. 5.

**Table 2:** Linguistic term set for criteria and sub-criteria

Linguistic term	Fuzzy value		
Very low(VL)	0	0	0.25
Low(L)	0	0.25	0.5
Moderate(M)	0.25	0.5	0.75
High(H)	0.5	0.75	1
Very High(VH)	0.75	1	1

The linguistic terms assigned by decision-makers to each criterion and sub-criterion for determining their importance are represents in Table 3. Tables 3 and 4 depict the importance allocated by decision-makers with respect to criteria and sub-criteria, respectively. Table 5 represents the ratings of alternatives assigned by decision-makers with respect to sub-criteria.

**Table 3:** Importance of criteria

Criteria	DM_1_	DM_2_	DM_3_	DM_4_	DM_5_
Economic	M	H	M	H	H
Environmental	VH	H	VH	H	H
Public Health	VH	VH	VH	VH	VH
Occupational Health	VH	VH	H	H	H
Technical	H	H	H	H	VH
Social	VH	H	VH	H	H

**Table 4:** Importance of sub-criteria

Decision maker	DM_1_	DM_2_	DM_3_	DM_4_	DM_5_
Sub-criteria					
CC*	H	M	M	H	H
OC	VH	H	H	H	H
WPPC	H	VH	H	H	H
SREI	VH	H	VH	H	H
WREI	VH	H	H	H	H
AREI	M	L	VL	M	L
Noise	M	M	L	L	VL
TOP	VH	H	VH	H	H
CP	VH	H	H	VH	H
MP	VH	VH	VH	VH	VH
RQMV	H	VH	H	H	M
RSC	H	VH	VH	H	H
Qual.SPW	H	H	VH	H	H
Quan.SPW	H	H	H	H	H
RWBD	VH	VH	VH	VH	VH
OHOF	VH	VH	VH	H	H
OHOI	H	VH	VH	H	H
REL	VH	VH	VH	H	H
WSR	H	VH	VH	H	H
EDPC	H	M	H	M	H
LA	H	M	M	M	H
NSO	M	M	L	VL	VL
NWTU	M	L	VL	L	VL
SDOC	M	M	L	VL	VL
EDOC	H	M	H	M	L
TC	M	M	L	VL	VL
DCTW	H	VH	H	H	H
NIV	H	VH	H	M	H
AUA	H	H	M	M	M
NPS	H	H	H	H	H
AEP	VH	H	VH	H	H
PAO	H	VH	VH	H	H
SOH	M	M	L	L	L
ATWS	VH	H	VH	H	H
EED	VH	VH	VH	H	H

**Table 5:** Ratings of the alternatives with respect to the sub-criteria (The full form of abbreviations was represented in Fig. 3)

Decision Maker	DM_1_				DM_2_				DM_3_				DM_4_				DM_5_			
Alternative	A_1_(PDDS)	A_2_(PBDW)	A_3_(PDWS)	A_4_(HDWU)	A_1_(PDDS)	A_2_(PBDW)	A_3_(PDWS	A_4_(HDWU)	A_1_(PDDS)	A_2_(PBDW	A_3_(PDWS)	A_4_(HDWU)	A_1_(PDDS)	A_2_(PBDW)	A_3_(PDWS)	A_4_(HDWU)	A_1_(PDDS)	A_2_(PBDW)	A_3_(PDWS)	A_4_(HDWU)
Sub-criteria																				
CC*	VL	VL	VL	VH	L	VL	L	VH	L	L	L	H	VL	VL	VL	VH	VL	VL	VL	VH
OC	VL	L	VL	VH	VL	M	VL	H	L	M	L	VH	L	H	VL	VH	VL	M	VL	H
WPPC	VL	H	M	L	VL	VH	L	VL	VL	VH	M	VL	L	VH	H	L	VL	VH	M	VL
SREI	L	VH	L	H	VL	VH	VL	VH	VL	VH	VL	M	L	H	L	H	VL	VH	VL	H
WREI	L	L	L	H	L	VL	L	VH	VL	VL	VL	H	L	VL	L	H	L	VL	L	VH
AREI	VL	VL	VL	VL	VL	VL	VL	L	VL	VL	VL	VL	VL	VL	VL	VL	VL	VL	VL	VL
Noise	VL	VL	VL	M	VL	VL	VL	M	VL	L	VL	H	VL	VL	VL	M	L	VL	L	M
TOP	L	VL	H	H	L	VL	VH	H	VL	VL	H	H	L	VL	H	H	L	L	H	M
CP	L	VL	VH	H	L	VL	H	M	L	VL	H	M	M	VL	VH	L	L	L	H	M
MP	VH	VL	VH	H	VH	VL	VH	M	H	VL	VH	H	VH	VL	H	H	VH	L	H	H
RQMV	VH	VH	H	L	VH	H	M	VL	H	VH	H	VL	H	H	M	VL	VH	H	M	VL
RSC	VH	L	VH	VH	VH	L	VH	H	VH	M	VH	H	H	L	VH	H	VH	VL	VH	H
Qual.SPW	VH	H	L	L	VH	VH	L	VL	VH	VH	VL	L	H	VH	L	L	VH	VH	L	L
Quan.SPW	L	M	M	VH	M	M	M	VH	M	M	L	H	L	H	L	H	L	M	M	M
RWBD	H	VL	H	M	M	VL	VH	M	H	L	H	L	H	VL	H	M	M	L	VH	M
OHOF	VH	L	VH	L	VH	L	VH	M	VH	M	VH	L	H	L	VH	L	H	L	H	M
OHOI	H	VL	H	M	H	VL	H	H	H	VL	H	H	H	VL	VH	H	M	L	H	M
REL	H	M	L	H	VH	H	VL	H	H	H	L	M	VH	VH	VL	M	M	VH	VL	H
WSR	H	VH	H	M	VH	H	VH	L	VH	VH	VH	M	VH	H	VH	L	H	M	H	L
EDPC	M	VL	M	VH	H	VL	H	VH	H	VL	H	VH	H	VL	H	VH	M	VL	M	H
LA	H	VL	VL	H	H	VL	VL	H	H	VL	VL	H	H	VL	VL	H	M	VL	VL	M
NSO	L	VL	L	VH	L	L	L	VH	VL	VL	VL	H	VL	VL	VL	VH	VL	VL	VL	H
NWTU	H	VL	L	VH	M	VL	VL	H	M	VL	VL	VH	H	VL	VL	H	H	VL	L	H
SDOC	M	VL	L	H	M	L	L	VH	L	L	VL	H	L	VL	L	VH	L	VL	L	H
EDOC	L	VL	L	VH	L	VL	VL	H	M	L	VL	VH	VL	VL	VL	VH	L	VL	VL	H
TC	L	VL	VL	VH	M	L	VL	H	M	L	VL	VH	L	VL	L	VH	L	L	L	VH
DCTW	VL	VL	VL	VH	VL	VL	VL	VH	VL	VL	VL	VH	VL	VL	VL	VH	VL	VL	VL	VH
NIV	VH	L	VH	VL	VH	L	VH	VL	H	M	H	L	VH	L	VH	VL	VH	L	VH	VL
AUA	H	H	H	L	H	H	VH	VL	VH	VH	VH	VL	VH	VH	VH	L	VH	VH	VH	L
NPS	L	L	L	VH	L	VL	L	VH	L	VL	L	VH	VL	VL	VL	H	L	VL	L	VH
AEP	M	VH	L	H	H	VH	L	M	M	VH	M	M	L	H	L	M	M	H	M	L
PAO	H	H	VH	M	M	H	VH	L	L	H	M	L	M	H	H	M	H	H	H	L
SOH	M	VL	M	H	L	L	L	H	L	VL	L	VH	M	VL	M	H	L	L	L	H
ATWS	M	L	M	VH	L	L	L	VH	M	M	M	VH	M	L	M	VH	M	L	M	H
EED	M	VH	VH	VL	L	VH	H	VL	M	VH	M	VL	L	H	H	L	M	H	H	L

## Results and discussion

Equations 4 and 5 were employed to aggregate the importance of criteria (see Table 3) and sub-criteria (see Table 4) and results were represented in Tables 6 and 7 for criteria and sub-criteria, respectively. The ratings of alternatives (see Table 5) were aggregated using Eq. 6 and results were shown in Table 8. It should be noted that in this study, the decision-makers were considered with equal weights *v*_*l*_. Thus ${v}_1={v}_2={v}_3={v}_4={v}_5=\frac{1}{5}$, as previously denoted by Dursun (2011a).

**Table 6:** Aggregated Importance weights of criteria

Criteria/Sub-criteria	Aggregated weights
Economic	(0.40, 0.50, 0.90)
Environmental	(0.60, 0.70, 1)
Public Health	(0.75, 0.80, 1)
Occupational Health	(0.60, 0.70, 1)
Technical	(0.55, 0.60, 1)
Social	(0.60, 0.70, 1)

**Table 7:** Aggregated Importance weights of sub-criteria

Sub-criteria	Aggregated weights
CC	(0.40, 0.50, 0.9)
OC	(0.55, 0.65, 1)
WPPC	(0.55, 0.65, 1)
SREI	(0.60, 0.70, 1)
WREI	(0.60, 0.70, 1)
AREI	(0.10, 0.25, 0.55)
Noise	(0.10, 0.30, 0.55)
TOP	(0.60, 0.70, 1)
CP	(0.60, 0.70, 1)
MP	(0.75, 0.80, 1)
RQMV	(0.50, 0.65, 0.95)
RSC	(0.60, 0.70, 1)
Qual.SPW	(0.55, 0.65, 1)
Quan.SPW	(0.50, 0.60, 1)
RWBD	(0.75, 0.80, 1)
OHOF	(0.65, 0.75, 1)
OHOI	(0.60, 0.70, 1)
REL	(0.65, 0.75, 1)
WSR	(0.60, 0.70, 1)
EDPC	(0.60, 0.70, 1)
LA	(0.60, 0.70, 1)
NSO	(0.60, 0.70, 1)
NWTU	(0.60, 0.70, 1)
SDOC	(0.60, 0.70, 1)
EDOC	(0.30, 0.50, 0.80)
TC	(0.10, 0.25, 0.50)
DCTW	(0.55, 0.65, 1)
NIV	(0.50, 0.60, 0.95)
AUA	(0.35, 0.50, 0.85)
NPS	(0.50, 0.60, 1)
AEP	(0.60, 0.70, 1)
PAO	(0.60, 0.70, 1)
SOH	(0.10, 0.30, 0.6)
ATWS	(0.60, 0.70, 1)
EED	(0.65, 0.75, 1)

**Table 8:** Aggregated ratings of alternatives with respect to sub-criteria

Sub-criteria	A_1_	A_2_	A_3_	A_4_
CC	(0.00, 0.10, 0.35)	(0.00, 0.05, 0.30)	(0.00, 0.10, 0.35)	(0.70, 0.95, 1)
OC	(0.00, 0.10, 0.35)	(0.25, 0.50, 0.75)	(0.00, 0.50, 0.30)	(0.65, 0.90, 1)
WPPC	(0.00, 0.05, 0.30)	(0.70, 0.95, 1)	(0.25, 0.50, 0.75)	(0.00, 0.10, 0.35)
SREI	(0.00, 0.01, 0.35)	(0.70, 0.95, 1)	(0.00, 0.10, 0.35)	(0.50, 0.75, 0.95)
WREI	(0.00, 0.02, 0.45)	(0.00, 0.05, 0.30)	(0.00, 0.20, 0.45)	(0.60, 0.85, 1)
AREI	(0.00, 0.00, 0.25)	(0.00, 0.00, 0.25)	(0.00, 0.00, 0.25)	(0.00, 0.05, 0.30)
Noise	(0.00, 0.05, 0.30)	(0.00, 0.25, 0.30)	(0.00, 0.05, 0.30)	(0.30, 0.55, 0.80)
TOP	(0.00, 0.20, 0.45)	(0.00, 0.05, 0.30)	(0.55, 0.80, 1)	(0.45, 0.70, 0.95)
CP	(0.05, 0.30, 0.55)	(0.00, 0.05, 0.30)	(0.60, 0.85, 1)	(0.25, 0.50, 0.75)
MP	(0.70, 0.95, 1)	(0.00, 0.05, 0.30)	(0.65, 0.90, 1)	(0.45, 0.70, 0.95)
RQMV	(0.65, 0.90, 1)	(0.60, 0.85, 1)	(0.35, 0.60, 0.85)	(0.00, 0.05, 0.30)
RSC	(0.70, 0.95, 1)	(0.05, 0.25, 0.5)	(0.75, 1, 1)	(0.55, 0.80, 1)
Qual.SPW	(0.70, 0.95, 1)	(0.70, 0.95, 1)	(0.00, 0.20, 0.45)	(0.00, 0.20, 0.45)
Quan.SPW	(0.01, 0.35, 0.60)	(0.30, 0.55, 0.80)	(0.15, 0.40, 0.65)	(0.55, 0.80, 0.95)
RWBD	(0.40, 0.65, 0.90)	(0.00, 0.10, 0.35)	(0.60, 0.85, 1)	(0.20, 0.45, 0.70)
OHOF	(0.65, 0.90, 1)	(0.05, 0.30, 0.55)	(0.70, 0.95, 1)	(0.10, 0.35, 0.60)
OHOI	(0.45, 0.70, 0.95)	(0.00, 0.05, 0.30)	(0.55, 0.80, 1)	(0.40, 0.65, 0.90)
REL	(0.55, 0.80, 0.95)	(0.55, 0.80, 0.95)	(0.00, 0.10, 0.35)	(0.40, 0.65, 0.90)
WSR	(0.65, 0.90, 1)	(0.45, 0.65, 0.75)	(0.65, 0.90, 1)	(0.10, 0.35, 0.60)
EDPC	(0.40, 0.65, 0.90)	(0.00, 0.00, 0.25)	(0.40, 0.65, 0.90)	(0.70, 0.95, 1)
LA	(0.45, 0.70, 0.95)	(0.00, 0.00, 0.25)	(0.00, 0.00, 0.25)	(0.45, 0.70, 0.95)
NSO	(0.00, 0.10, 0.35)	(0.00, 0.05, 0.30)	(0.00, 0.10, 0.35)	(0.65, 0.90, 1)
NWTU	(0.40, 0.65, 0.90)	(0.00, 0.00, 0.25)	(0.00, 0.10, 0.35)	(0.60, 0.85, 1)
SDOC	(0.05, 0.20, 0.35)	(0.00, 0.10, 0.35)	(0.00, 0.20, 0.45)	(0.60, 0.85, 1)
EDOC	(0.05, 0.25, 0.50)	(0.00, 0.05, 0.30)	(0.00, 0.05, 0.30)	(0.65, 0.90, 1)
TC	(0.10, 0.35, 0.60)	(0.00, 0.15, 0.40)	(0.00, 0.10, 0.35)	(0.70, 0.95, 1)
DCTW	(0.00, 0.00, 0.25)	(0.00, 0.00, 0.25)	(0.00, 0.00, 0.25)	(0.75, 1, 1)
NIV	(0.70, 0.95, 1)	(0.05, 0.30, 0.55)	(0.70, 0.95, 1)	(0.00, 0.05, 0.30)
AUA	(0.65, 0.90, 1)	(0.65, 0.90, 1)	(0.70, 0.95, 1)	(0.00, 0.15, 0.40)
NPS	(0.00, 0.20, 0.45)	(0.00, 0.05, 0.30)	(0.00, 0.20, 0.45)	(0.70, 0.95, 1)
AEP	(0.25, 0.50, 0.75)	(0.65, 0.90, 1)	(0.10, 0.35, 0.60)	(0.25, 0.50, 0.75)
PAO	(0.30, 0.55, 0.8)	(0.50, 0.75, 1)	(0.55, 0.80, 0.95)	(0.10, 0.35, 0.60)
SOH	(0.10, 0.35, 0.60)	(0.50, 0.10, 0.35)	(0.10, 0.35, 0.60)	(0.55, 0.80, 1)
ATWS	(0.20, 0.45, 0.70)	(0.05, 0.30, 0.55)	(0.20, 0.45, 0.70)	(0.70, 0.95, 1)
EED	(0.15, 0.40, 0.65)	(0.65, 0.90, 1)	(0.50, 0.75, 0.95)	(0.00, 0.10, 0.35)

Normalized ratings of alternatives with respect to sub-criteria were computed using Eq. 8 which is based on the linear scale transformation approach (results were not shown). Then, aggregate performance ratings (APRs) of alternatives with respect to sub-criteria are calculated by Eq. 9 (results were not shown). Eq. 9 was applied to aggregate the sub-criteria values to criteria level according to the findings of Karsak (2002). Normalized APRs were calculated by using Eq. 10 and results are illustrated in Table 9, in which, 0 implies the worst value and 1 represents the best value.

**Table 9:** Normalized the aggregated performance ratings

Criteria/Sub-criteria	Aggregated weights
Economic	(0.40, 0.50, 0.90)
Environmental	(0.60, 0.70, 1)
Public Health	(0.75, 0.80, 1)
Occupational Health	(0.60, 0.70, 1)
Technical	(0.55, 0.60, 1)
Social	(0.60, 0.70, 1)

The weighted distances from ideal solutions (*D*_i_^*^) and anti-ideal solutions (*D*_*i*_^−^) were computed using Eq. 11 and 12, respectively. Then, the proximity of the alternatives to the ideal solution (*Ω*_i_^*^) was calculated by using Eq. 12. The results of the *D*_i_^*^, *D*_*i*_^−^ *and Ω*_i_^*^ values are presented in Table 10.

**Table 10:** Ranking of the drinking water alternatives

Alternative	*D*_*i*_^∗^	*D*_*i*_^−^	*Ω*_*i*_^∗^	Rank
A_1_: Public Desalinated Distribution System (PDDS)	2.131	3.346	0.611	1
A_2_: PET Bottled Drinking Water (PBDW)	2.212	3.405	0.606	2
A_4_: Household Desalinated Water Units (HDWU)	2.279	3.482	0.604	3
A_3_: Private Desalinated Water Suppliers (PDWS)	2.384	3.01	0.558	4

After sorting the alternatives according to the magnitude of *Ω*_i_^*^values, the following ranking order was achieved:

$$ {A}_1\kern0.5em >\kern0.5em {A}_2\kern0.5em >\kern0.5em {A}_4\kern0.5em >\kern0.5em {A}_3 $$

As can be inferred from Table 10 the Public Desalinated Distribution System (A_1_) is the best alternative as drinking water source for Qom population.

Abrishamchi and co-workers (2004) denoted a small potable water network (less than 30 km) with public valves (water standpipes) at several points across the city of Zahidan. They considered the “Extension of the small drinking water distribution network with public standpipes” as an alternative to supply the drinking water needs of population.

Public Desalinated Distribution System (PDDS) has several benefits such as simple operation of treatment facilities and ease of health inspection process. Now, more than 180 km of potable water network has been constructed in the city of Qom which have connected to 260 public valve (water standpipes) and supply more than 4500 cubic meter of desalinated water per day [29]. The only noticeable problem dealing with the PDDS is the low extension of distribution system which tends to handle the water containers from public valves to houses by people.

Jafaripour estimated that over 36000 houses in Qom use the Household desalinated water units (HDWU) which cover more than 15 % of all population. Based on the findings of Jafaripour, more than 1000 m^3^ of brine water and up to 550 discarded filter are produced by using of Household desalinated water units (HDWU) [30].

Yari reported that 24 Private desalinated water suppliers (PDWS) are operated in the city of Qom. Their results showed that the chemical characteristics of potable water produced by PDWS could not meet the national standard criteria. Also, transferring the water containers by vendees is the other constraint of PDWS. Purchased water containers may stored in homes for a long time in uncontrolled health condition [31].

More than 18 various brands of PET Bottled Drinking Water (PBDW) are sold in the retails of Qom city [32]. Noticeable merits of PBDW are Chemical and biological acceptable quality which serve as an alternative beside the other water supply system. High price and lack of coverage for all population, in the other hand, are the essential drawbacks of PBDW.

A significant factor that should be considered in the judgment process of purchasing high-tech equipment is the level of dependency to the foreign suppliers. A more appropriate strategy is to encourage the use of the alternative technologies available within the country. Hence, except for the household desalinated water units (HDWU), the other alternatives could not obtain higher levels of linguistic terms by decision-makers for SDOC and EDOC sub-criteria.

Considering the occupational and public health criteria independent of the environmental and technical criteria significantly improved the precision of the results.

## Conclusions

An efficient analysis was performed by applying the evaluation criteria and their associated sub-criteria on a hierarchical structure. Thirty five sub-criteria associated with six criteria were structured in a multi-level hierarchy and the decision processes allowed the decision-makers to employ linguistic concepts, and thus, decreased the cognition problems during the evaluation process.

In this study, hierarchical distance-based fuzzy multi-criteria group decision making (DBF –MCDM) approach was presented to avoid the problems that may occurred when the classical decision-making approaches are employed for evaluating the water supply alternatives.

New arrangement of criteria and sub-criteria was proposed in this study. Traditionally, four criteria including financial, environmental, technical, and social aspects have been proposed in similar works. Using a new hierarchy containing the public health and occupational health aspects as the independent criteria enabled the decision-making process to assign more effective evaluations.

System and equipment dependency to other countries (SDOC and EDOC) were added to the technical aspects as sub-criteria for obtaining a state of compatibility with the socioeconomic condition which restrict the level of dependency on the foreign companies.

The DBF–MCDM method proposed in this research is a simple approach that can be used for similar environmental management issues only with some modifications.
